# Green Light Photo Vaporization of the Prostate Versus Minimal Invasive Surgical Techniques (MIST): A Patient-Centered Shared Decision Making Approach

**DOI:** 10.7759/cureus.114972

**Published:** 2026-08-22

**Authors:** Mohamed Abouelenein, Osman Ermis, Noman Ali Ghazanfar, Atınç Tozsin, Theodore Patel, Omer Onur Cakir, Sanjith Gnanappiragasam, Kawa Omar, Edward Bass, Francesca Kum

**Affiliations:** 1 Urology, King’s College Hospital NHS Foundation Trust, London, GBR; 2 Urology, İstanbul Avcilar Murat Kölük State Hospital, Istanbul, TUR; 3 Urology, Silopi State Hospital, Sirnak, TUR; 4 Surgery, King’s College Hospital NHS Foundation Trust, London, GBR; 5 Urology, King's College Hospital NHS Foundation Trust, London, GBR; 6 Urology, Maidstone and Tunbridge Wells Hospitals, Maidstone, GBR

**Keywords:** benign prostate enlargement, greenlight laser photoselective vaporization of the prostate (gll pvp), lower urinary tract symptoms (luts), minimal invasive surgical techniques, patient assessment, peri-operative medicine, prostate enlargement, shared-decision making

## Abstract

Background

Benign prostatic hyperplasia (BPH) is a highly prevalent cause of lower urinary tract symptoms (LUTS) in the aging male population. Photoselective vaporization of the prostate (PVP) and minimally invasive surgical treatments (MISTs) are established surgical alternatives to traditional resection, each with distinct mechanisms of action and clinical profiles.

Objective

The study aims to compare the perioperative parameters and functional outcomes of PVP versus MISTs in patients with moderate-to-severe LUTS secondary to BPH.

Methods

This was a retrospective observational cohort study conducted at a single tertiary center, including patients treated between October 2023 and January 2026. Ethics approval was obtained from the Research Ethics Committee at King’s College Hospital NHS Foundation Trust, London, UK (approval reference number: 25/LO/8780); the requirement for individual written consent was waived given the retrospective, fully anonymized nature of the data. After removal of duplicate and incomplete records, 99 patients treated with PVP and 64 patients treated with MIST (water vapor thermal therapy (WVTT), n=58; prostatic urethral lift (PUL), n=5; temporarily implanted nitinol device (i-TIND), n=1) were analyzed. Baseline and three-month postoperative maximum urinary flow rate (Q-max), post-void residual volume (PVR), and International Prostate Symptom Score (IPSS) were compared within each cohort using paired-samples t-tests (available-case analysis, restricted to patients with both baseline and follow-up values recorded), and between cohorts using Welch's independent-samples t-test on change scores.

Results

Both cohorts showed statistically significant within-group improvement across all evaluated parameters. In the PVP cohort, Q-max improved by a mean of 6.55 ml/s (n=24, 95% CI 2.54-10.57, p=0.003), PVR decreased by 222.6 ml (n=28, 95% CI −356.1 to −89.2, p=0.002), and IPSS decreased by 8.13 points (n=15, 95% CI −15.83 to −0.43, p=0.040). In the MIST cohort, Q-max improved by 3.74 ml/s (n=33, 95% CI 1.17-6.30, p=0.006), PVR decreased by 68.9 ml (n=42, 95% CI −97.3 to −40.5, p<0.001), and IPSS decreased by 11.65 points (n=23, 95% CI −15.40 to −7.90, p<0.001). Direct between-group comparison of change scores showed a significantly greater reduction in PVR with PVP (difference of −153.7 ml, p = 0.028) but no significant between-group difference in Q-max change (p = 0.231) or IPSS change (p = 0.391). Mean baseline prostate volume was larger in the PVP group (61.6 vs. 50.0 cc, p < 0.001).

Conclusions

Both PVP and MIST produced statistically significant improvement in urinary flow, post-void residual volume, and symptom burden at three months. PVP demonstrated a significantly greater reduction in PVR, consistent with more complete anatomical resolution of obstruction, but showed no significant advantage over MIST in Q-max or IPSS improvement in this cohort. Treatment selection should be individualized to patient priorities, procedural setting, and anticipated recovery profile, rather than assuming uniform PVP superiority across all functional domains. Larger prospective studies with complete outcome capture are needed, including evaluation of sexual function, which was not assessed in this cohort.

## Introduction

Lower urinary tract symptoms (LUTS) due to benign prostatic hyperplasia (BPH) constitute a common clinical entity in the aging male population, with a substantial impact on quality of life (QoL) and a considerable economic burden on health-care systems ​[1]. Their prevalence increases with age, affecting a large proportion of men beyond the seventh decade of life ​[1-3]. For most patients, management begins with lifestyle measures and pharmacotherapy, while surgical intervention is generally reserved for those who fail or do not tolerate medical treatment or who develop complications such as refractory urinary retention, recurrent infection, bladder stones, or hematuria ​[1]. Although monopolar transurethral resection of the prostate (M-TURP) was historically regarded as the reference surgical technique for benign prostatic obstruction (BPO), bipolar TURP (B-TURP) has emerged as the contemporary standard of care due to its more favorable safety profile ​[1-4]. Currently, both techniques serve as the benchmark against which newer minimally invasive interventions are evaluated ​[1]. The risks associated with conventional resection, however, including bleeding, transurethral resection (TUR) syndrome, and the broader perioperative morbidity, have driven the development of safer and less invasive technologies, and the range of procedures available for LUTS/BPO has widened considerably in recent years ​[2,5].

Photoselective vaporization of the prostate (PVP) with the 532-nm green-light laser is among the most established of these alternatives ​[5,6]​. Because this wavelength is selectively absorbed by hemoglobin, adenomatous tissue is vaporized with a hemostatic effect that limits intraoperative bleeding ​[6,7]. In terms of clinical efficacy, reflected in the improvement of the International Prostate Symptom Score (IPSS) and the maximum urinary flow rate (Q-max), PVP has yielded results comparable to and, in randomized settings, non-inferior to those of TURP, while offering shorter catheterization, a reduced length of hospital stay, and the possibility of treating higher-risk patients who remain on antiplatelet or anticoagulant therapy ​[5,8,9]. 

Accordingly, the European Association of Urology (EAU) guidelines recommend offering 532-nm laser vaporization of the prostate as an alternative to TURP in men with moderate-to-severe LUTS/BPO and a prostate volume of 30-80 mL ​[1]. At the same time, several longer-term series have suggested that reoperation or retreatment rates after PVP may be somewhat higher than those reported after conventional TURP ​[5,10]. 

With the further aim of reducing surgical morbidity and, in particular, of preserving sexual function, a group of newer treatments commonly referred to as minimally invasive surgical treatments (MISTs) has become increasingly popular ​[2,11,12]. These include the prostatic urethral lift (PUL), convective water vapor thermal therapy (WVTT), the temporarily implanted nitinol device (i-TIND), prostatic artery embolization (PAE), image-guided robotic waterjet ablation (Aquablation), trans-perineal interstitial laser ablation (TPLA), and paclitaxel-coated balloon dilatation (Optilume), among others ​[1]. These procedures can generally be performed under local anesthesia, often permit same-day discharge, and are associated with high rates of preservation of sexual function, including antegrade ejaculation and erectile function as commonly assessed by the five-item International Index of Erectile Function (IIEF-5) ​[11-13]. Their effect on objective functional parameters such as Q-max and post-void residual (PVR) volume is, however, generally more modest than that achieved with resection or ablative techniques, and medium-to-long-term retreatment rates tend to be higher; together, these represent the principal limitations of the minimally invasive approach ​[1,2,11].

Direct functional comparisons between PVP and MISTs remain scarce in the literature. PVP is most often compared against TURP or HoLEP, while individual MIST modalities are usually compared against one another or against TURP. Because PVP and MISTs are frequently considered for overlapping patient populations despite differing mechanisms, a direct comparison of functional outcomes may help clarify their respective roles in clinical decision-making. The aim of this study was to compare perioperative parameters and functional outcomes of PVP versus MIST for BPO at our institution to inform patient selection and clinical counseling.

## Materials and methods

Study design and ethical approval 

This was a retrospective observational cohort study conducted at King's College Hospital NHS Foundation Trust, London, UK, including patients treated between October 2023 and January 2026. The study protocol was reviewed and approved by the Research Ethics Committee at King's College Hospital (approval reference number: 25/LO/8780). Given the retrospective nature of the study and the use of anonymized routine clinical data, the requirement for written informed consent was waived by the ethics committee, as data were collected retrospectively. All data were fully anonymized prior to analysis, and no identifying patient information appears in this manuscript. The study was conducted in accordance with the principles of the Declaration of Helsinki. Prior to their index procedure, all patients had received detailed clinical counseling regarding the risks, benefits, and procedural mechanisms of both surgical modalities as part of routine clinical care. 

Patient selection and criteria 

Male patients aged 50-85 years with moderate-to-severe LUTS secondary to BPH who had failed conservative medical therapy were eligible. Patients were divided into a PVP (GreenLight laser; Boston Scientific, Marlborough, MA, USA) cohort and a MIST cohort (WVTT, PUL, or i-TIND). Exclusion criteria comprised prior surgical intervention for bladder outlet obstruction (BOO); need for a concomitant/adjuvant procedure during the index surgery; clinical or biochemical suspicion of prostate cancer; neurological deficit identified as the primary cause of LUTS; unwillingness to participate; or comorbidities precluding standard same-day discharge. After exclusion of duplicate patient records and procedures outside the PVP/MIST definitions (e.g., isolated TURP), the final analytic cohorts comprised 99 PVP patients and 64 MIST patients (WVTT n=58, PUL n=5, i-TIND n=1).

Preoperative evaluation and treatment allocation and postoperative follow-up

Baseline assessment included medical history, physical examination, and digital rectal examination. IPSS and QoL scores were recorded using the standardized instrument of Barry et al. (1992) [14]; this scale is in the public domain, developed under the auspices of the American Urological Association, and is reported for research purposes without modification. No separate license or permission letter is required, which we confirm to the editor accordingly. Prostate volume was measured by transrectal or transabdominal ultrasound. Q-max and PVR were measured by clinic-based uroflowmetry. Treatment allocation to PVP or MIST was based on patient preference following counseling. Follow-up assessment, including repeat uroflowmetry and IPSS/QoL scoring, was performed at three months postoperatively using identical instruments.

Statistical analysis 

Continuous variables (Q-max, PVR, IPSS) are expressed as means with standard deviations. Within-cohort change from baseline to three-month follow-up was assessed using the paired-samples t-test (two-tailed), restricted to patients with both a recorded baseline and follow-up value for the given metric (available-case analysis); degrees of freedom, 95% confidence intervals, and Cohen's dz (standardized mean difference for paired data) are reported alongside each t-statistic. A between-cohort comparison of mean change scores was performed using Welch's independent-samples t-test, which appropriately accounts for unequal variances between groups; between-group Cohen's d is reported. A p-value <0.05 was considered statistically significant. Analyses were performed in IBM SPSS Statistics software, version 27 (IBM Corp., Armonk, NY, USA). 

## Results

**Figure 1:**
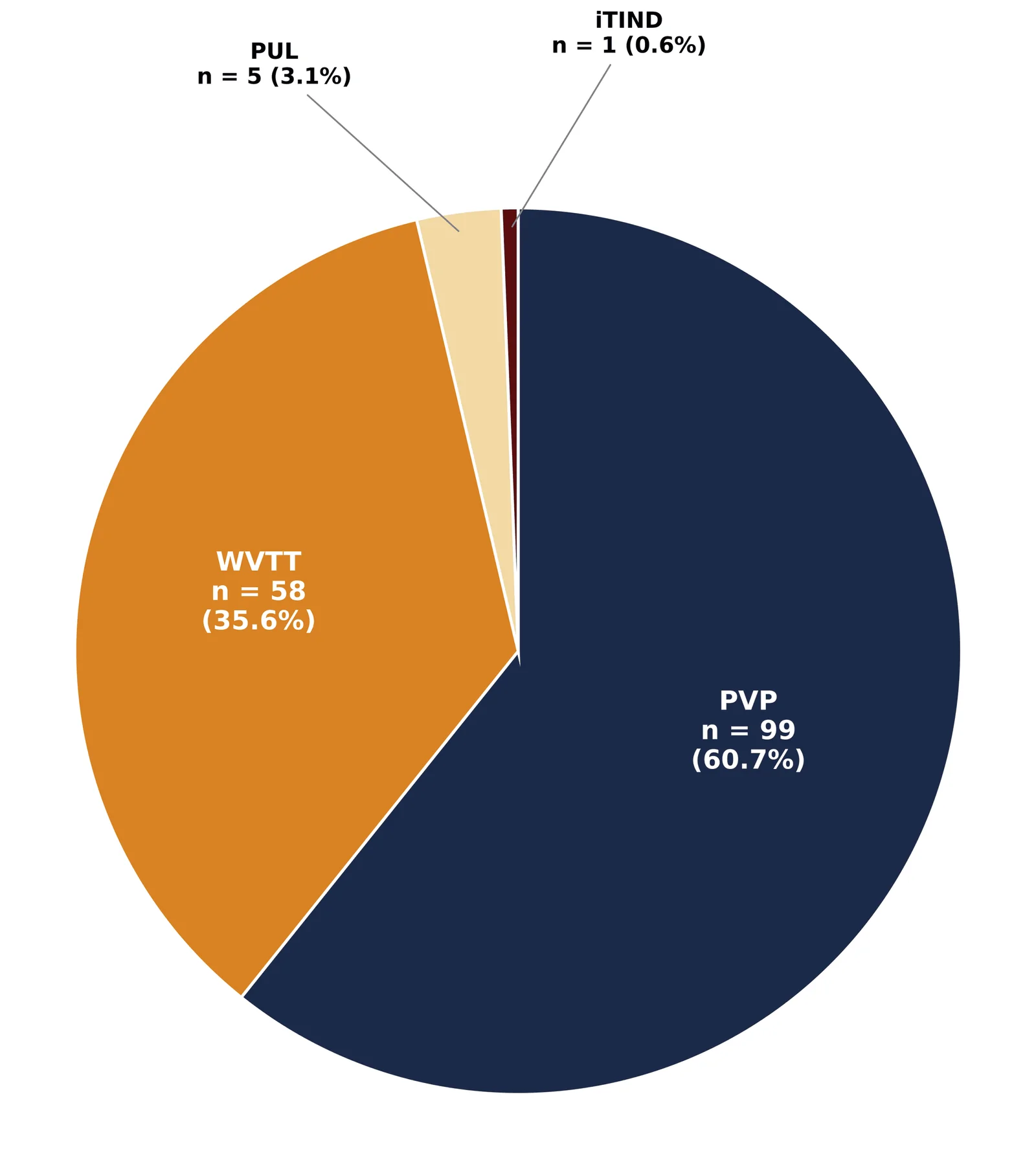
Distribution of patients by treatment: PVP and MIST subtypes Abbreviations: i-TIND, temporarily implanted nitinol device; MIST, minimally invasive surgical treatment; PUL, prostatic urethral lift; PVP, photo-selective vaporization of the prostate; WVTT, convective water vapor thermal therapy.

A total of 163 unique patients were analyzed: 99 in the PVP cohort and 64 in the MIST cohort (WVTT n=58, PUL n=5, i-TIND n=1). Mean baseline prostate volume was significantly larger in the PVP cohort than the MIST cohort (61.6 ± 22.8 cc (n=95) vs. 50.0 ± 18.0 cc (n=63); Welch's t(155.6)=3.58, p<0.001). Analysis of demographic and baseline clinical data revealed a mean age of 65.7 years for the PVP group and 68.9 years for the MIST group.  The mean baseline prostate volume was 63.24 cc in the PVP cohort and 50.62 cc in the MIST cohort (Figure 1). 

Within-cohort functional outcomes

All three parameters improved significantly from baseline, with moderate-to-large effect sizes (dz 0.59-0.69). The IPSS result, while significant, rested on the smallest available sample (n=15) and the widest relative confidence interval and should be interpreted more cautiously than the Q-max and PVR findings (Table 1).

**Table 1:** Preoperative and postoperative functional outcomes in the PVP cohort Note: IPSS and QoL scores were assessed using the standardized instrument originally developed by Barry et al. (1992) [14]. Abbreviations: CI, confidence interval; IPSS, International Prostate Symptom Score; PVR, post-void residual volume; Qmax, maximum urinary flow rate; QoL, quality of life.

Metric	n	Preoperative mean	Postoperative mean	Mean change	95% CI	t (df)	p	Cohen's dz
Q-max (ml/s)	24	9.95	16.50	+6.55	2.54 to 10.57	3.38 (23)	0.003	0.69
PVR (ml)	28	283.39	60.75	−222.64	−356.09 to −89.20	−3.42 (27)	0.002	−0.65
IPSS	15	23.00	14.87	−8.13	−15.83 to −0.43	−2.27 (14)	0.040	−0.59

MIST also produced significant improvement across all parameters, with the IPSS effect size (dz = −1.34) notably larger than any effect observed in either cohort - indicating that symptom relief with MIST was not only statistically significant but substantial in magnitude, despite the cohort's less ablative mechanism (Table 2).

**Table 2:** Preoperative and postoperative functional outcomes in the MIST cohort Note: IPSS and QoL scores were assessed using the standardized instrument originally developed by Barry et al. (1992) [14]. Abbreviations: CI, confidence interval; IPSS, International Prostate Symptom Score; MIST, minimally invasive surgical treatment; PVR, post-void residual volume; Qmax, maximum urinary flow rate; QoL, quality of life.

Metric	n	Preoperative mean	Postoperative mean	Mean change	95% CI	t (df)	p	Cohen's dz
Q-max (ml/s)	33	10.77	14.51	+3.74	1.17 to 6.30	2.97 (32)	0.006	0.52
PVR (ml)	42	96.07	27.17	−68.90	−97.34 to −40.47	−4.89 (41)	<0.001	−0.76
IPSS	23	27.91	16.26	−11.65	−15.40 to −7.90	−6.45 (22)	<0.001	−1.34

Between-cohort comparison

The two cohorts are distinguishable only on PVR-the parameter most directly tied to physical tissue removal-while Q-max and IPSS changes overlap substantially between treatments. This pattern suggests PVP's advantage is specific to anatomical obstruction relief rather than a generalized functional superiority. PVP showed a significantly greater reduction in PVR compared with MIST (p=0.028). No statistically significant between-group difference was observed for change in Q-max (p=0.231) or IPSS (p=0.391); the point estimate for IPSS change numerically favored the MIST cohort, though this difference was not statistically significant (Table 3).

**Table 3:** Comparison of mean functional improvements between the PVP and MIST cohorts Note: All functional parameters were assessed at baseline and at the three-month postoperative follow-up. IPSS and QoL scores were assessed using the standardized instrument originally developed by Barry et al. (1992) [14]. Abbreviations: IPSS, International Prostate Symptom Score; MIST, minimally invasive surgical treatment; PVR, post-void residual volume; PVP, photoselective vaporization of the prostate; Qmax, maximum urinary flow rate; QoL, quality of life.

Metric	PVP means change (n)	MIST means change (n)	Difference (PVP−MIST)	Welch	p	Cohen's d
Q-max (ml/s)	+6.55 (24)	+3.74 (33)	+2.81	1.22	0.231	0.34
PVR (ml)	−222.64 (28)	−68.90 (42)	−153.74	−2.31	0.028	−0.67
IPSS	−8.13 (15)	−11.65 (23)	+3.52	0.88	0.391	0.32

## Discussion

Summary of the main findings 

Both PVP and MIST produced statistically significant improvement in Q-max, PVR, and IPSS from baseline to three-month follow-up. When change scores were compared directly between cohorts, PVP demonstrated a significantly greater reduction in PVR, but no significant advantage over MIST was found for improvement in Q-max or IPSS. While both modalities successfully alleviate bladder outlet obstruction, the magnitude of functional improvement was distinctly higher in the laser vaporization arm. These results suggest that while minimally invasive approaches are effective, the immediate physical debulking achieved through tissue vaporization yields more robust objective and subjective functional recovery during follow-up ​[6,8]. 

PVP efficacy, prostate volume, and operative time 

The significantly larger reduction in PVR with PVP is consistent with its mechanism of direct tissue debulking, and aligns with the larger mean baseline prostate volume in this cohort (61.6 vs. 50.0 cc, p<0.001) [8]. This supports the interpretation that PVP provides more definitive relief of anatomical obstruction, independent of whether flow rate and symptom score improvements are statistically distinguishable from MIST in this sample.

Functional outcomes of minimally invasive treatments 

The MIST cohort's mechanism-dependent, less ablative approach [2,11] was nonetheless associated with statistically and clinically meaningful improvement across all three outcomes, including the largest effect size observed in this study for IPSS (dz = −1.34). This suggests that, contrary to an assumption of uniformly modest MIST efficacy, symptom-score improvement with MIST in appropriately selected patients can be substantial. Consequently, improvements in objective parameters such as PVR and Qmax remain more conservative compared to laser ablation. This finding is consistent with current clinical guidelines, which acknowledge that while MISTs offer favorable perioperative safety profiles, their objective functional results are generally more modest ​[1]​. Furthermore, recent data in the literature emphasize that this less aggressive tissue modification is associated with a progressively increasing risk of surgical reintervention, particularly at the three-year and five-year post-procedural milestones ​[1,2,11,12]​. 

Clinical advantages of MISTs

The principal advantage of the MIST cohort in our study was significantly shorter operative time and minimal perioperative morbidity, facilitating an efficient perioperative course [2, 13]. Sexual function preservation is a recognized advantage of MISTs in the broader literature: the avoidance of direct thermal or mechanical injury to the peri-prostatic neurovascular bundle and bladder neck is thought to allow MISTs to maintain erectile and ejaculatory function while still delivering acceptable symptom relief [3,12]. This is strongly supported by a recent meta-analysis of over 11,000 patients by Cahill et al. (2026), which found that MISTs successfully preserve and, in some cases, improve erectile and ejaculatory function compared with traditional BPH surgeries [12]. Erectile function was not directly assessed in our own dataset, so this advantage should be regarded as supported by the wider literature rather than by our results.

Context within the literature

Large registry studies provide robust data on retreatment and complication rates but often lack detailed functional metrics such as longitudinal IPSS, QoL, or uroflowmetry data [2,10]. This study contributes real-world, head-to-head functional data, while highlighting the practical challenge of incomplete outcome capture in retrospective cohorts, a limitation that should inform the design of future prospective comparisons.

Study limitations 

Our study is not without limitations. Its retrospective design introduces an inherent selection bias, as evidenced by the baseline differences between the cohorts. Specifically, the larger mean prostate volume in the PVP group indicates a natural clinical tendency among surgeons to favor ablative techniques for larger glands, which inherently contributes to the longer operative times observed in this arm. Furthermore, the MIST cohort was heterogeneous, comprising convective WVTT, PUL, and iTIND procedures, which rely on different mechanisms of action and may independently influence clinical outcomes. Long-term prospective trials are needed to further validate these findings. 

## Conclusions

In this retrospective cohort, both PVP and MIST produced statistically significant improvements in Q-max, PVR, and IPSS at the three-month follow-up. Direct between-group comparison showed PVP achieved significantly greater reduction in post-void residual volume, consistent with its ablative mechanism and the larger prostate volumes treated in that cohort, but no statistically significant advantage over MIST in Q-max or IPSS improvement. Treatment selection should therefore be individualized based on prostate volume, obstruction severity, and patient priorities, rather than an assumption of uniform PVP superiority across all functional domains. Larger, prospective studies with complete and protocolized outcome capture are warranted.
